# Anti-cancer peptide-based therapeutic strategies in solid tumors

**DOI:** 10.1186/s11658-022-00332-w

**Published:** 2022-04-09

**Authors:** Mohsen Karami Fath, Kimiya Babakhaniyan, Maryam Zokaei, Azadeh Yaghoubian, Sadaf Akbari, Mahdieh Khorsandi, Asma Soofi, Mohsen Nabi-Afjadi, Hamidreza Zalpoor, Fateme Jalalifar, Ali Azargoonjahromi, Zahra Payandeh, Armina Alagheband Bahrami

**Affiliations:** 1 Department of Cellular and Molecular Biology, Faculty of Biological Sciences, Kharazmi University, Tehran, Iran; 2 Department of Medical Surgical Nursing, School of Nursing and Midwifery, Iran University of Medical Sciences, Tehran, Iran; 3 Department of Food Science and Technology, Faculty of Nutrition Science, Food Science and Technology/National Nutrition and Food Technology Research Institute, Shahid Beheshti University of Medical Sciences, Tehran, Iran; 4 Department of Veterinary Medicine, Beyza Branch, Islamic Azad University, Beyza, Iran; 5 Department of Exercise Physiology, Central Tehran Branch, Islamic Azad University, Tehran, Iran; 6 Faculty of Pharmacy, Mashhad University of Medical Sciences, Mashhad, Iran; 7 Department of Biotechnology, Faculty of Pharmacy, Mashhad University of Medical Sciences, Mashhad, Iran; 8 Department of Physical Chemistry, School of Chemistry, College of Sciences, University of Tehran, Tehran, Iran; 9 Department of Biochemistry, Faculty of biological science, Tarbiat Modares University, Tehran, Iran; 10 American Association of Kidney Patients, Tampa, FL USA; 11 Shiraz Neuroscience Research Center, Shiraz University of Medical Sciences, Shiraz, Iran; 12 Network of Immunity in Infection, Malignancy & Autoimmunity (NIIMA), Universal Scientific Education & Research Network (USERN), Tehran, Iran; 13 Shiraz University of Medical Sciences, Shiraz, Iran; 14 Department Medical Biochemistry and Biophysics, Division Medical Inflammation Research, Karolinska Institute, Stockholm, Sweden; 15 Department of Biotechnology, School of Advanced Technologies in Medicine, Shahid Beheshti University of Medical Sciences, Tehran, Iran; 16 School of Medicine, Jiroft University of Medical Sciences, Jiroft, Iran

**Keywords:** Peptide-based strategies, Tumor-homing peptides, Drug delivery, Targeted delivery

## Abstract

**Background:**

Nowadays, conventional medical treatments such as surgery, radiotherapy, and chemotherapy cannot cure all types of cancer. A promising approach to treat solid tumors is the use of tumor-targeting peptides to deliver drugs or active agents selectively.

**Result:**

Introducing beneficial therapeutic approaches, such as therapeutic peptides and their varied methods of action against tumor cells, can aid researchers in the discovery of novel peptides for cancer treatment. The biomedical applications of therapeutic peptides are highly interesting. These peptides, owing to their high selectivity, specificity, small dimensions, high biocompatibility, and easy modification, provide good opportunities for targeted drug delivery. In recent years, peptides have shown considerable promise as therapeutics or targeting ligands in cancer research and nanotechnology.

**Conclusion:**

This study reviews a variety of therapeutic peptides and targeting ligands in cancer therapy. Initially, three types of tumor-homing and cell-penetrating peptides (CPPs) are described, and then their applications in breast, glioma, colorectal, and melanoma cancer research are discussed.

## Background

Selective delivery of chemotherapeutic agents to cancer cells to ensure the effectiveness of treatment while minimizing damage to healthy tissue is one of the most important aspects of cancer therapy [1–3]. Expression or overexpression of a particular group of proteins or receptors on the cell surface is one of the biological features of tumor cells [4–6]. Activation of these receptors leads to deregulated signaling, decreased cell apoptosis, and increased cell proliferation. Moreover, these oncogenic alterations could promote invasion of tumor cell into surrounding tissues. Targeting these tumor-specific receptors selectively leads to targeted drug delivery [7, 8]. Monoclonal antibodies, antibody fragments, polypeptides, small compounds, and various types of ligand have been developed for tumor targeting [9, 10]. Antibodies are one of the most common ligands in clinics for tumor targeting. Antibodies can effectively target antigens delivering anticancer drugs directly to tumor tissues. Some limitations of antibodies for clinical use are slow diffusion into tumor tissue, limited in vivo stability, and high production costs [7, 11, 12]. On the other hand, antibodies have difficulty penetrating target tissues because of their enormous size. They can also be toxic in the liver, bone marrow, and spleen and cause immunogenicity by attaching to the reticuloendothelial system [13]. The majority of patients treated with immune checkpoint inhibitors such as monoclonal antibodies suffer from drug-induced immune-related adverse events (irAEs). Accordingly, irAEs are expected to appear in patients treated with anti-CTLA-4 (60–85%), anti-PD-1 (16–37%), and anti-PD-L1 (12–24%). Furthermore, as with the use of the combination of anti-CTLA-4 and anti-PD-1 or anti-PD-L1, the frequency and severity of irAEs were higher than with single immune checkpoint inhibitor monoclonal antibody treatment. Approximately 60% of patients who used combination therapy may suffer severe side effects, including autoimmune inflammation in the heart and the nervous system [14, 15].

Extensive research across the therapeutic spectrum of peptides indicates their potential application in the treatment and diagnosis of multiple cancers, that is, peptide therapy. Tumor-specific peptides are a good choice to selectively target tumor-specific receptors. Tumor-targeted peptides have higher tumor/tissue penetration than antibodies, and their chemical modification and production facilitate increased stability and pharmacokinetics [13]. These peptides are used in imaging, cancer diagnosis, and targeted drug delivery. In addition, particular peptides can be engineered to attack cancer cells and prevent tumor progression.

Peptides could be used in a variety of ways to treat cancer [16, 17]. For example, they can deliver treatments to cancer cells, simulate natural proteins to enhance or inhibit signal transduction, or intercede therapeutic transport across a barrier [18]. Peptides can also be rapidly resynthesized by fully automated methods, and peptide screening methods are very powerful and adaptable. Moreover, research shows that certain peptides can cross the blood–brain barrier and have effects on the central nervous system.

Production of peptides is less complex than that of proteins and antibodies. They can be easily synthesized in various cells, coupled to different substances, and modified in different manners. Because of their simpleness and easy transport, peptides have a longer shelf life [18].

In this review, we introduce three categories of tumor-homing and cell-penetrating peptides (CPPs) and summarize the recent advances of peptides in the diagnosis, prognosis, and prediction of breast, glioma, colon, and melanoma cancer application.

## Types of peptide and their function in cancer

A peptide is a linear chain of amino acids (AA) consisting of fewer than 50 AA that are stabilized by disulfide bonds. They are molecularly intermediate between small molecules and macromolecules, but have sufficient biochemical and therapeutic differences. Rational methods can be used to design peptides that specifically bind to and modulate protein interactions of interest, such as oncogenic proteins [16]. There are three main sources of peptide production: (a) bioactive or natural peptides, (b) engineered peptides created using recombinant or genetic libraries, and (c) peptides derived from chemical libraries [17]. Low-molecular-weight peptides have a relatively high affinity for entering tumor tissues. It would be possible to synthesize them chemically, which would be a cost-effective cancer treatment [19–21].

It is possible to obtain peptides from a variety of natural sources [22]. Peptides that are antitumor or anticancer can be plant-derived, such as *Ganoderma lucidum* polysaccharide peptide (Gl-PP), which is anti-angiogenic [23], or animal-derived, such as atrial natriuretic peptide [24].

A variety of peptides derived from animal proteins, such as angiotensin and growth-inhibitory peptide (GIP) derived from α-fetoprotein, also exhibit significant anticancer properties [25, 26]. Marine source peptides such as jaspamide and somocystinamide A (mediate apoptosis) and Aplidin (causes cell cycle arrest) have shown potent antitumor effects [27]. Microbial peptides, including mycobacteria-derived muramyl dipeptide (MDP), FK565, and *Streptomyces*-derived bestatin have shown anticancer properties [28].

Increasing the half-life of peptides is possible by preventing their degradation by blood proteases through blocking the C- and N-termini of peptides or forming cyclically shaped peptides [29].

Peptides that are used in the targeted delivery of medications are classified into three categories: peptides that enter the tumor, peptides that target abnormal intracellular signaling pathways, and peptides that enter cells [30].

Tumor-homing peptides bind to molecules that are overexpressed or particularly expressed on the cancer cell’s surface. Some of these peptides may stimulate or inhibit signaling pathways in cancer cells or tumor tissue by binding to them. Oncogenic signaling pathways, which control cancer cell activity, escape of apoptosis, and tumor cell proliferation, are identified as targets for peptides targeting abnormal cellular signaling pathways. Thus, well-designed peptides and peptide derivatives can result in improved tumor treatment.

Small peptides known as cell-penetrating peptides (CPPs) are derived from viral, insect, or mammalian proteins that can penetrate the cell membrane. Peptides like these can be covalently attached to a variety of drug carriers and used for drug delivery, as well as incorporated into imaging agents, nanoparticles, liposomes, oligonucleotides, and specific molecular targets in cancer cells [30].

In the following sections, we highlight peptides’ potential as promising anticancer agents and the role they play in improving the delivery of therapeutics across the membrane of cancer cells.

## Peptide-based strategies for targeted breast cancer

Breast cancer, as an invasive cancer, starts in the breast tissue and spreads to the milk-producing lobules and ducts. On the basis of its clinical features, four forms of breast cancer have been identified: ductal carcinoma in situ (DCIS), invasive ductal carcinoma (IDC), inflammatory breast cancer (IBC), and metastatic breast cancer. Owing to its aggressive nature, IDC can be fatal and accounts for more than 80% of breast cancer cases [29, 31–33].

Although, in most cases, the actual cause of breast cancer is unknown, age, alcohol consumption, obesity, and estrogen exposure are all known risk factors. Moreover, breast cancer is associated with a number of genetic, environmental, and epigenetic risk factors. Mutations in tumor suppressor genes 1 and 2 (BRCA1 and BRCA2), which are involved in repairing damaged DNA, are among the genetic factors studied. Mutations in BRCA1 or BRCA2 increase the risk of breast and ovarian cancer by 5- and 10–30-fold, respectively [34–36].

As breast cancer cells become more resistant to anticancer drugs, researchers are looking for new ways to treat them. Here, peptide therapy has emerged as a viable treatment option for solid tumors, especially breast cancer. Peptides are attractive treatments because of their precise attachment to the surface of tumor cells, low molecular weight, and low toxicity to normal cells [37, 38].

### Therapeutic peptides as inhibitors of transport agents in breast cancer cells

Transcription factors (TFs) regulate the timing and rate of gene expression in specific cells. In breast cancer cells, a variety of TFs are important for cell survival, growth, metastasis, and division. Therefore, peptide therapy by targeting these factors may be a good approach for treating breast cancer [39, 40].

MYC proto-oncogene, basic helix–loop–helix (bHLH) transcription factor, regulates growth, proliferation, metabolism, differentiation, and apoptosis in cells. Three transcription factors are encoded by the Myc gene family: c-Myc, L-Myc, and N-Myc. The helical leucine zipper domain of the protein MYC binds to DNA. It forms a heteromer set with the MYC-associated X (Max) factor, which binds to E-boxes in gene promoters [39]. During the G1 phase of cell proliferation, the MYC–MAX complex is a cell cycle motility reducer [41].

According to studies, MYC is overexpressed in 30–50% of high-grade cancers and about 15% of breast tumors [42]. Thus, the targeting of MYC or its downstream signaling pathways may be a viable treatment option for breast cancer. Drager and Mullen have developed a short peptide (H1-S6A.F8A) from the HI3 region that interacts with c-Myc and prevents it from binding to DNA. This peptide has higher helicity thanks to two exchanged amino acids (S6A and F8A). The binding of H1 peptides to the tetrameric c-Myc-92 is the mechanism of inhibition [43].

Bidwell et al. fused the H1-S6A.F8A peptide to a modified elastin-like polypeptide that could penetrate cells and play a role as a transport vehicle for a short peptide in a separate study (Pen-ELP-H1).

Pen-ELP-H1 disturbs the c-Myc nuclear localization and inhibits its transcriptional activity as demonstrated by immunofluorescence and RT-PCR. The growth of human MCF-7 cancer cells was also slowed by the peptide [44]. The peptide transition temperature was 39 °C, which makes it an ideal candidate for thermal targeting [45]. Compared with a thermal-insensitive control polypeptide, hyperthermia almost doubled the antiproliferative activity of Pen-ELP H1. Thus, conjugated peptides combined their inhibitory effects with heat, which may have synergistic benefits for treating localized cancers such as breast cancer [46].

### Therapeutic peptides as cytolytic agent in breast cancer cells

Cytolytic peptides (5–30 AAs) have amphiphilic and cationic characteristics, allowing them to penetrate cell membranes and kill cells. They are produced in various plants and animals and cause nonspecific lysis [47]. These nonspecific cuts are a limitation to their clinical application. Thus, peptides with cytolytic effects are usually combined with other peptides capable of directly recognizing cancer cells [48].

Another important disadvantage of cytolytic peptides is their nonspecific cytotoxicity, which was discussed in a study by Zhao et al. They synthesized two lytic peptides, Ur11 and Uk14, and combined them with an anion peptide with a matrix metalloproteinase-sensitive cleavage linker. Coated peptides were used against the MDA-MB-231 breast cancer cell line. The results indicate that matrix metalloproteinases can destroy the cytolytic membrane of cancer cells [48].

Furthermore, Zhong et al. synthesized a membrane-lytic peptide that could degrade type 1 metalloproteinases with a ring separated by a membrane and use the peptides against three various cells that express various metalloproteinases. They found that, when cyclic peptides split by metalloproteinases become linear, and threshold cells in particular are destroyed by the enzyme [49].

### Therapeutic peptides as tumor suppressor proteins in breast cancer cells

Tumor-suppressing proteins arrest cell progression from phase G1 to S. several of these proteins play a critical role in preventing CDKs. Within mammalian cells, numerous kinases are expressed in G1-phase mitotic cells, including the complexes Cdk4/cyclin-D, Cdk6/cyclin-D, Cdk2/cyclin-E, Cdk2/cyclin A, and Cdk1/cyclin A, which play key roles in progressing the cells from phase G1 to phase S. Tumor-suppressing therapeutic peptides are synthetic peptides that regulate CDKs in the cell cycle and may be effective therapies in breast cancer treatment as they arrest G1-phase cells [50]. These peptides are less likely to cause side effects and can be removed quickly [51]. This peptide significantly suppresses the growth of different cancer cells, such as MCF-7, and prevents the cells from entering the S phase [52].

Proliferating cellular nuclear antigen (PCNA) inhibits G1 and G2 cell cycle phases and DNA replication. The p21-derived tumor suppressor peptide from the C-terminal region can inactivate PCNA, which is necessary for cell proliferation [53].

PCNA can be targeted and inactivated by p21-derived peptides, including 139–164 AA and 144–151 AA [54]. These peptides cause inhibition of cell cycle progression and cell proliferation [55].

Recent studies have indicated that novel therapeutics can be created using tumor-suppressive peptides in place of existing chemotherapeutics that are ineffective at targeting metastases. In a study by Jalota Badhawar et al., a TAT-SMAR1-derived chimeric peptide (p44) considerably activated p53 by phosphorylating serine 15, via activating p21 protein and inhibiting cell cycle progression [56].

Oncosuppressive properties of the p53 pathway make it an attractive target. Following the emergence of MDM2 as a p53 inhibitor, pharmacological disruption of the interaction between these two proteins has been explored to reactivate p53 in human tumors that express wild-type TP53. As a result, different classes of molecules have been developed that are capable of interfering with the formation of the MDM2/p53 complexes (such as Nutlin) [57–59]. Pellegrino et al. developed a method of interfering with the heterodimerization of MDM2/MDM4 complexes to inhibit their activity. They discovered that the binding of a peptide mimicking the MDM4 C-terminus tail to MDM2 impairs MDM2-mediated ubiquitination of p53 and promotes p53-dependent transcription and oncosuppressive activities [57].

White et al. identified a peptide targeting the C-terminal domain of BRCT2 in breast cancer [60]. BRCT2 has been linked to DNA damage signaling [60].

### Therapeutic peptides as stimulants of death in breast cancer cells

Various peptides have been discovered to trigger breast cancer cell death in studies. KCCYSL peptide and peptides containing KCCYSL sequence are specific peptides for human epidermal growth factor receptor 2 (HER2) overexpressed in breast cancer. For greater efficiency, attachment of the nuclear localization sequence peptide (NLP) to the KCCYSL peptide leads to translocation of the peptide to the nucleus of HER2^+^ cells, resulting in cell death. Therefore, KCCYSL-based peptides can be used for imaging of HER2-positive tumors with k_d_ of 295 nM [61].

Zhang et al. also designed and synthesized a transformable peptide with the ability to self-assemble into micelles in aqueous solution and transform into nanofibrils inhibiting HER2 dimerization and subsequent apoptosis of cancer cells [62].

Another receptor with increased levels in progressive cancerous breast cells is estrogen receptor α (ERα), which can be targeted for peptide-based cancer therapy. In this regard, proteolysis-targeting chimeras (PROTACs) containing a ligand for target protein and a recognition motif for E3 ubiquitin ligase recruitment are candidates for ERα degradation and inhibition of cancerous cell growth. Dai et al. attached proteolysis-targeting chimeras (PROTACs) to lactam cyclic peptide as ERα-binding ligand, 6-aminocaproic acid as a linker, and a hydroxylated structure for recruiting E3 ligase known as 1–6 compound. They noted that 1–6 compound has a strong effect on MCF-7 cell toxicity with IC_50_ of ~ 9.7 μM via inducing ERα degradation [63].

The pro-apoptotic Bcl-2 proteins are divided into two subgroups that include multivalent proteins, such as Bak, Bax, and Bok [64].

On the basis of the structural properties of Bcl-2 proteins, numerous peptides have been designed to induce apoptosis of cancer cells [65].

For instance, normally, anti-apoptotic Bcl-2 factor causes cell death by inhibiting pro-apoptotic members of the Bcl-2-family in the mitochondria. Bcl-2 factors also inhibit IP3 receptors (IP3R) in the endoplasmic reticulum and increase survival of cancerous cells.

Kerkhofs et al. targeted the IP3 receptor (IP3R) channels and induced the downstream pro-apoptotic Ca^2+^-signaling pathway via synthesizing a peptide named Bcl-2 IP3 receptor disrupter-2 (BIRD-2) mimicking the structure of the BH4 domain of Bcl-2. The peptide disrupted the IP3R–Bcl-2 interaction, leading to the death of the cancer cells [66].

Suarez and colleagues used Phage Display Deep L to select specific peptides for HER2/neu and verified them in the breast cancer cell line SKBR3. These peptides were shown to destroy breast cancer cells. They also found that these peptides lowered BCL-XL and d MCL-1 while increasing Bax [67].

Voltage-dependent anion channel 1 (VDAC1)-derived peptides target anti-apoptotic proteins and induce cell death in cancer cells but not in noncancer cells, as shown by several studies [68]. Chen and colleagues discovered that mitochondrial damage triggers apoptosis. They used folic acid cation (FA) and triphenylphosphonium (TPP) to create a dual-targeting pro-apoptotic peptide [69]. FA enhances receptor-mediated peptide uptake and has the ability to transport peptides into the mitochondria of cancer cells [69].

Zhao et al. investigated the toxicity of combined lytic and anionic peptides via matrix-sensitive metalloproteinase-binding compounds in the MDA-MB-231 breast cancer cell line. Because of the nonspecific activation of lytic peptide, they discovered that, when the peptide enters the cell line, it specifically cleaves secreted metalloproteinases, breaking the peptide bond [48].

Advantages of pro-apoptotic peptides include high solubility in water, low immunogenicity, low-cost synthesis, and efficient chemical modification. Nevertheless, some barriers such as limited tumor cell uptake, low selectivity, poor tumor permeability, and low plasma stability need to be overcome. Various strategies such as intracellular delivery via intracellular pathway, ligand-mediated tumor targeting, and nanoparticle-based drug delivery systems may be discussed to address these problems [70].

### Antimicrobial peptides as cytolytic agent in breast cancer cells

Antimicrobial peptides are new anticancer medications made by eukaryotic and prokaryotic organisms that can kill cancer cells. These peptides have been replaced by other medications, particularly antibiotics, owing to their beneficial characteristics. The permeability of antimicrobial peptides is quite low. They have a strong affinity with minimum medication interference and vast diversity [71].

Improving specificity, destroying the ability to transport to tumors, maintaining low toxicity, and stabilizing serum are key challenges when employing antimicrobial peptides to treat breast cancer. These limitations, like those of other therapeutic peptides, can be overcome by employing a variety of molecular and nanoparticle-based drug delivery systems (DDS) [28].

TTPs have the ability to bind to indicators such as receptors, which are abundant in tumor cell membranes. These peptides include RGD, which contains an Arg–Gly–Asp sequence. In ovarian [72] and breast cancers [73], this sequence can detect integrins 3 and 5.

A defensive peptide targets breast cancer and suppresses metastasis, according to Papu et al. (2006). They also discovered that the peptide targets phosphatidylserine, which selects cancer cells. Their findings suggest that disrupting the barrier could provide a new therapeutic technique for halting tumor growth and preventing metastasis [74]. The effect of cationic antimicrobial peptides from the pleurocidin family, NRC-03 and NRC-07, on breast cancer cells was studied by Hilchi et al. They showed the ability of these peptides to destroy breast cancer cells, even drug-resistant forms. Noncancerous cell lysis, on the other hand, was minimal to nonexistent [75].

Wang et al. showed that temporin-1CEa peptide, derived from dermal secretions, has rapid cytotoxic activity in MDA-MB-231 and MCF-7 breast cancer cells [76].

In addition, they investigated the suitability of L-K6 antimicrobial peptide to target the cell surface of MCF-7 cells. They found an interaction of L-K6 and MCF-7 at high levels due to its negatively charged phosphatidylserine.

At the same time, they showed that localization of L-K6 through clathrin-independent micropinocytosis, without disturbing the cell surface in the cells, causes nuclear damage and cell death, without significant disruption of the cytoskeleton and mitochondrial dysfunction [77].

They found that the cytotoxic effect of this chimeric protein was enhanced by two peptide effects, as it had a greater effect than p28 alone compared with normal cells after 48 h of treatment [78]. Owing to the high number of negatively charged phosphatidylserine on the surface of cancer cells, cationic amphipathic peptides appear to be suitable anticancer agents in the treatment of cancer [78].

In another study, Zhang et al. targeted β-catenin, an important factor in WNT signaling, which is accumulated in the cytoplasm of cancer cells, causing cell growth. In this study, they applied antimicrobial peptide SKACP003 against β-catenin in three breast cancer cell lines: MCF-7, MDA-MB-453, and MDA-MB-231. The results indicated that SKACP003 induced dose-dependent cytotoxicity in all three cell lines via hydrogenic interaction of the peptide with β-catenin [62].

### Peptide vaccines to treat breast cancer

Cancer therapy with peptide vaccines is a new and attractive immunotherapeutic approach. Peptide vaccines are designed to elicit highly targeted immune system responses, thus avoiding allergic or reactive sequences [79]. They create and spread tumor T cells to control or kill cancer cells.

Advantages of peptide vaccines include the following: (a) easy and cost-effective synthesis and purification; (b) safety and stability; (c) simple administration of peptide in clinical environment; (d) effective at inducing the CD8/CD4 T-cell response in the human body. However, some disadvantages include: (a) rapid degradation by serum or tissue peptidases; (b) poor safety; (c) tolerance to short peptides that may bind directly to MHC in nonprofessional antigen-presenting cells (APCs); (d) transient or weak immune responses [80]. Cancer antigens are delivered by CD8^+^ or CD4^+^ T cells by APCs with proteolytic processing for MHC class I and II, respectively [81].

Several peptide vaccines have been designed for breast, prostate, colorectal, pancreatic, and melanoma cancers, some of which have passed phase I and II clinical trials with promising results [31, 82, 83]. Dillon et al., in a phase I clinical trial, evaluated a vaccine containing nine breast-cancer-associated peptides (MHC I restricted) from MAGE-A1, 3A3, and AA10 proteins, CEA, NY-ESO-1, and HER2/neu along with a TLR3 agonist, Poly-ICLC, and an adjuvant derived from tetanus toxin. The results of ELIspot assay indicated that toxicity in reaction to or stimulation of injection site, fever, and fatigue symptoms were negligible in almost all patients. Excipients and adjuvant peptides induce a mild immune activation: CD8^+^ T-cell responses were reported in 4 of 11 patients [84].

Studies have also shown that the use of granulocyte–macrophage colony-stimulating factor (GM-CSF) along with E75, known as NeuVax, is very effective in cancer [85].

Clifton et al. tested NeuVax in two clinical trials [86]. They found that HER2/neu was expressed at different levels in patients. The vaccine was well tolerated, stimulated the immune response, and had mild toxicity. Based on the results, NeuVax, also called nelipepimut-S, is in phase III of testing [86, 87]. The results of the phase III trial indicate common side effects in all groups [88].

## Peptide-based strategies for targeted glioma cancer

Glioblastoma is considered an aggressive and fatal type of brain cancer. It presents as differentiated anaplastic cells surrounded by brain tissue necrotic areas [89, 90]. Common approaches of glioblastoma therapy encompass radiotherapy and chemotherapy. Noteworthily, sometimes such methods of treatment are not achievable because the tumor cells have certain features such as being capable of growing, invading, and metastasizing frantically [91]. Moreover, some tumor cells that resist radiotherapy and chemotherapy can change to the secondary form of glioblastoma lesions and cause tumor recurrence. Along with resistance to treatment in some patients with glioblastoma, the inability to cross the blood–brain barrier (BBB) is deemed another flaw of such methods of treatment [30].

Selvarathinam et al. presented a pH/reduction-sensitive carboxymethyl chitosan nanogel (CMCSN) modified by targeting peptide angiopep-2 (ANG) and loading with doxorubicin (DOX) named DOX-ANG-CMCSN; hence, anticancer drugs penetrates the BBB via ANG [92].

It is of note that glioma cells have the ability to express tumor-specific biomarkers on the surface of their cells, as do vascular networks related to glioma cells [93], such as transferrin [94], insulin [95], and glucose transporters (e.g., GLUT-1) [96]. Peptides recognizing these receptors are used to deliver chemotherapy [97, 98], imaging probes, siRNA, DNA, and nanoparticles to the brain tumors. Although such peptides are unable to pass through the BBB, it is a prevailing notion that some use receptor-mediated transcytosis [99].

These types of peptide—being used as deliverer—are classified into three categories: tumor-homing peptides, peptides targeting aberrant cellular signaling pathways, and cell-penetrating peptides (CPPs) [100].

The short CPPs are derived from mammalian, viral, or insect proteins that can cross the cell membrane. Further, these peptides can be joined with different drug carriers covalently and used for imaging agents, targeted drug delivery, nanoparticles, oligonucleotides, and liposomes to target specific molecules within cancer cells.

Owing to the heterogeneous nature of glioblastoma disease and its complex pathogenesis, including mutations/alterations in some key cellular signaling pathways, the use of peptides to target oncogenes or signaling molecules is a promising therapeutic approach in this disease treatment. Specific molecular signaling pathways in glioblastoma could be inhibited by therapeutic peptides and can lead to anticancer activity [30].

VDAC1, which is overexpressed in glioblastoma cells, plays an essential role in cell energy metabolism. It regulates mitochondria that mediate apoptosis through interaction with anti-apoptotic proteins and protects glioblastoma cells against cell death [101, 102].

Shteinfer-Kuzmine et al. established that VDAC-1 based peptides conjugated to CPP or transferrin receptor suppress tumor growth strongly in the orthotopic glioblastoma mouse model [103].

This report indicates that the NF-κB pathway could enhance angiogenesis, tumor growth, survival, and cell proliferation [104]. Friedmann-Morvinski et al. designed a peptide that targets NEMO, blocking NEMO interaction with the IKK (IκB)-kinase complex, thus inhibiting the activity of NF-κB [105].

It has been shown that NBD treatment results in a decrease in tumor growth rate in mouse and human glioblastoma models and extends survival time from 30 to 50 days in mice. This evidence is reliable confirmation that the NF-κB pathway can be deemed a promising target for glioblastoma treatment.

Because c-Myc has an important role to play in controlling tumorigenesis, cell growth, proliferation, and apoptosis, peptides targeting c-Myc could be useful in preclinical evaluations. Deregulated expression of c-Myc has been shown to be associated with malignancy. Also, c-Myc expression is higher in glioma cancer stem cells than in non-stem cells [106, 107]. To block the c-Myc pathway, a peptide derived from helix 1 (H1) of the HLH region of c-Myc [43] was linked with a CPP and thermoresponsive elastin-like polypeptide (ELP) [108]. The CPP-ELP-H1 reduced the tumor volume by 80% and doubled the mean survival time.

### Cell-penetrating peptides in glioma

Regarding the impermeability of the BBB and cell membrane to therapeutic macromolecules (of large size and low permeability), the incorporation of CPPs, which increase the crossing of the BBB and plasma membrane in peptide-based drug delivery systems, can be a clever solution. Studies on CPP-based drug delivery systems have indicated their effectiveness in multiple models of cancer, including glioblastoma [109].

Recently, investigations indicated that protegrin-derived SynB1 CPPs, and Antennapedia homeodomain-derived penetratin (antp), could increase the doxorubicin delivery (an anticancer drug) across the BBB, which indicates their ability to treat glioblastoma [110, 111]. The nonspecificity of CPP in transporting different cargoes to cells is a concern. To optimize the CPP-derived therapeutics’ specificity, they could be combined with tumor-homing ligands or other systems of targeted delivery to ensure selective and effective drug delivery.

### Tumor-homing peptides in glioma

According to genomics and proteomics studies in glioblastoma, the expression of specific proteins on the surface of cancer cells is increased compared with normal brain tissues [112].

Glioblastoma cells overexpress a receptor for natural ligands known as low-density lipoprotein receptor-related protein (LRP). It attaches to human melanotransferrin (p97), receptor-associated protein (RAP), lactoferrin [113], and synthetic peptides, including peptide analog of ApoE3 [114] and angiopep-2 (ANG) [115].

ANG can be conjugated to a variety of carriers to deliver small molecular drugs and genetic materials to the brain. ANG1005, ANG conjugated to paclitaxel (PTX), has previously been investigated in clinical trials for malignant glioma recurrence [116].

**Fig. 1:**
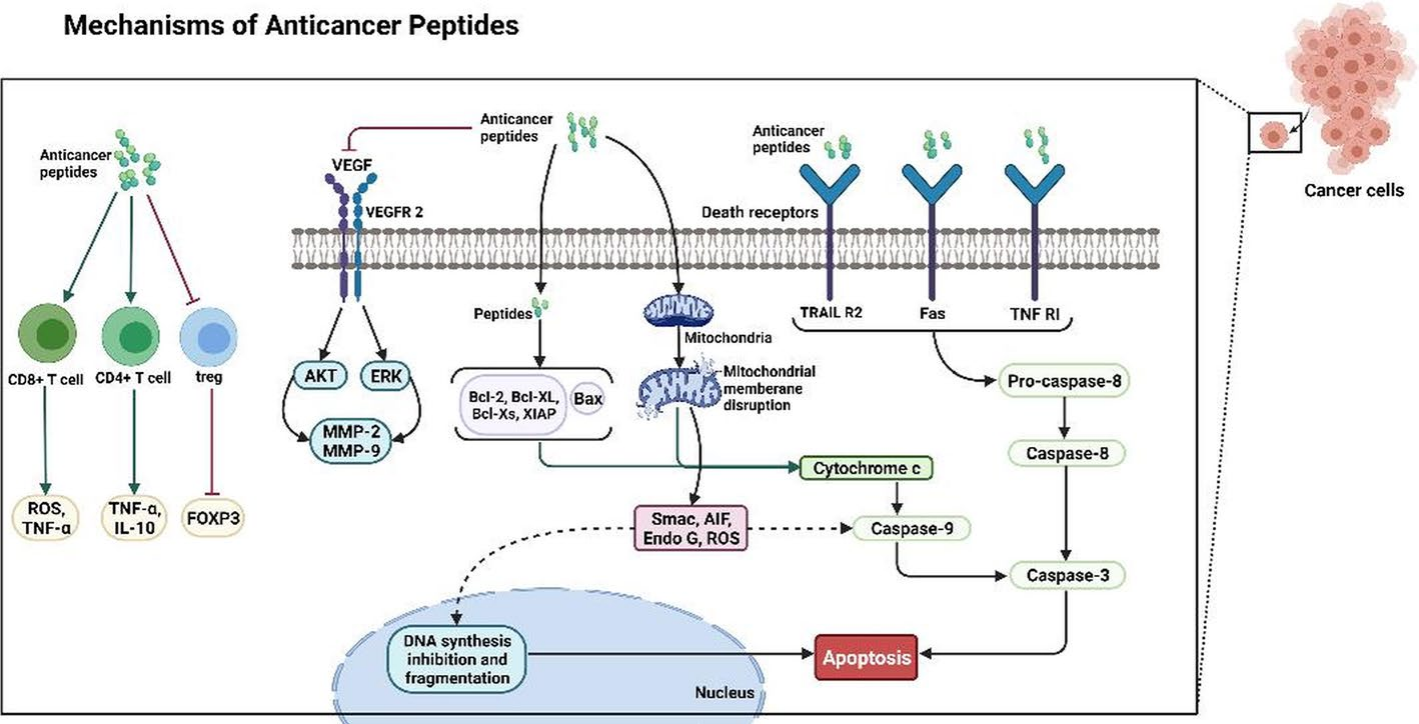
Anticancer peptide mechanisms of action: mitochondrial-associated pathways and death-receptor-induced pathways

Another class of glioma-specific peptides was discovered while investigating glioma-specific chloride channels. The expression of these channels is higher in high-grade tumors than in low-grade tumors in glioblastomas. Chlorotoxin (36 AAs), derived from the venom of the scorpion Leiurus quinquestriatus, is one of these peptides that bind specifically to these channels [117] (Fig. 1).

### Glioblastoma-targeting Nona peptide

#### MDGI/FABP3 targeting peptide—CooP

Glioblastoma or grade IV glioma is an aggressive type of cancer that mostly occurs in adults [118, 119]. With the urgent need to find novel tumor vascular targeting agents, Hyvönen et al. used the in vivo phage display screen to identify a linear glioblastoma-targeting Nona peptide (CooP: CGLSGLGVA)[93] in which MDGI/FABP3 plays a vital role as the CooP-interacting partner. The expression of MDGI is crucial for glioma cells and is grade dependent in human brain tumor specimens [93, 120]. The discovery of CooP peptide led to the enhancement of the delivery of chemotherapy and nanoparticles to gliomas and other types of solid tumor that results from the overexpression of MDGI. CooP homing to a diverse intracranial aneurysm animal model of glioma such as the mouse astrocyte-derived HIF-1α-deficient (HIFko) glioma has been reported [121].

In vivo murine models revealed that radiolabeled (indium, ^111^In) CooP peptide can be utilized for homing to glioblastoma-bearing mice in SPECT-CT for diagnostic imaging [93]. The CooP peptide as a tumor-targeting carrier in peptide–drug conjugates offers a number of benefits, including enhanced targeting capacity of the drug and cell penetration [122]. As a result, the drug conjugate showed efficacy in the treatment of glioblastoma compared with the free drug in our intracranial HIFko glioma model [93].

#### Transferrin receptor targeting peptides—T7/T12

Transferrin receptor (TfR) is a cell-membrane-associated glycoprotein whose expression is regulated by the iron-regulatory protein system. TfR mediates the delivery of iron to the blood–brain barrier and intracellular trafficking of the iron-binding protein transferrin. TFR1 and TFR2 are two subtypes of TFR [123]. While both peptides are broadly used to target several types of cancers, T12 exhibited higher affinity toward the target, leading to more successful results compared with the T7 peptide. Furthermore, T12 peptide was inefficient at transporting conjugated compounds across the BBB in an M13 phage display library trace [124].

#### LRP targeting peptides—Angiopep-2

LRP, including LRP-1 and LRP-2, is a ubiquitous endocytic cell membrane receptor that binds various ligands and controls the permeability of the BBB [96, 125].

As a result, LRP functions as a receptor and can also be used in brain-targeting drug delivery. As suggested, LRP-1 is a multifunctional receptor expressed on glioblastoma cells and glioma-associated human endothelial cells. Classical endocytic receptor LRP2 expresses on the apical surface of absorptive epithelia tissue. Accordingly, LRP2 plays a key role in endocytosis and subsequent intracellular transport.

In addition, the extracellular ligand-binding domain of LRP2 binds to various large macromolecules, including lipoprotein lipase, apolipoproteins E and B, and albumin. In addition, polyethylene glycol (PEG) copolymer coated with superparamagnetic iron oxide nanoparticles was able to enhance the retention time in circulation. In vivo MR imaging of glioblastoma showed improved signal by using dual-targeting nanoprobe with lower signal intensity [96, 125].

### Interleukin-4 receptor targeting peptide—AP

According to homology sequence search, the AP binding sequence (84KRLDRN8) motif correlates with the interleukin 4 receptor (IL-4R) [126]. The plasma membrane IL-4R receptor is highly expressed in human malignant glioma cell lines, suggesting that CRKRLDRNC plays a key role in glioma targeting.

By the use of U87MG in vitro models, Park et al. attained promising results with peptide-conjugated AP for targeted doxorubicin delivery for therapeutics and glioma-targeted imaging [127].

## Peptide-based strategies for targeted colorectal cancer

One of the most frequent types of cancer worldwide is colorectal cancer (CRC). Early detection of CRC is associated with a better prognosis [128, 129]. Peptides are commonly used in the diagnosis of CRC. Indeed, they could be employed as CRC biomarkers. For example, in a recent clinical study, increased levels of human neutrophil peptides 1–3 (HNP1–3) were detected in CRC tissue, notably in Dukes’ stages C and D [128] In addition, high levels of peptide C in the blood in men indicate a higher risk of adenoma, according to Comstock et al. [125].

The role of peptides in CRC treatment, in addition to their role in CRC detection, is noteworthy. Atrial natriuretic peptide (ANP) is a cardiovascular-derived peptide hormone known for its antiproliferative properties that is considered a potential treatment for CRC [130].

Tumor growth is dependent on neovascularization. Neovasculature is a promising target for cancer therapy. TCP-1, a peptide discovered by Li et al., specifically targets blood arteries in tumor tissues [131]. This peptide can also transport fluorescein and drugs for imaging and apoptosis in CRC. According to research, TCP-1 can deliver anticancer drugs to CRC tissue while avoiding normal tissue. Therefore, it can be effective in treating CRC [131].

In a study by Wang et al., the F56-peptide-fused nanoparticles loaded with vincristine (F56-VCR-NP) were able to target both neovasculature and primary lesions of lung metastases, leading to neovasculature apoptosis and CRC tissue destruction [132].

Additionally, combined peptides have been well examined for CRC treatment. Inoda et al. indicated that the combination of three peptides, Cep55/c10orf3 402, Cep55/c10orf3 193, and Cep55/c10orf3 283, was effective in HLA-A24-positive CRC [133].

Hazama et al. recently reported the potential of a “peptide cocktail” for treatment of patients with CRC. Their report showed that the mean overall survival time in patients receiving the peptide cocktail improved compared with the control group [134]. In their subsequent research, the increased level of IL-6 due to the peptide vaccine suggested that IL-6 could serve as a good indicator in patients who received the peptide vaccine [135].

The use of a seven-peptide cocktail vaccine with oral chemotherapy by Okuno et al. showed improved results in patients with metastatic CRC and led to longer survival time compared with the control group [136].

These data confirm the efficacy of peptides in the diagnosis and treatment of CRC, as well as the combined use of peptides, including drug-bound peptides, and the use of the “peptide cocktail” vaccine.

## Peptide-based strategies for targeted melanoma cancer

Gallic acid has effective antiviral, antifungal, and antioxidant activity, although at high temperatures it has low solubility and is unstable. To overcome these limitations, Soe et al. fused RGD to gallic acid and synthesized galloyl-RGD. The experimental results showed decreased levels of intracellular cyclic adenosine monophosphate (cAMP) and inhibition of cAMP-responsive element-binding protein (CREB) phosphorylation [137]. Several studies have shown that cyclic RGD, such as the cyclic polymeric RGD-containing peptides, compared with linear RGD or monomeric cyclic types, exhibits strong binding activity toward integrin targets [138, 139].

In other studies, the M2PEP sequence has been used to target TAM and has shown positive results. Whole nanoparticles containing LyP-1, IMT, and anti-CTLA-4 showed the greatest effect. A 50% reduction in tumor volume compared with free anti-CTLA-4 indicates the potential of peptide-based nanoparticles to improve the clinical standard [140, 141].

The neuropilin-1 (Nrp1) receptor was first discovered as a potential T_reg_ marker by Broder et al. in 2004 [142]. Nrp1 is now essential for T_reg_ function. The expression of Nrp1 in T_reg_ is associated with the expression of FoxP3, which suggests its role as a mediator of the immunosuppressive phenotype [143].

Delgofe et al. showed the role of Nrp1 in maintaining the stability of T_reg_, as instability of this population via Nrp1 upregulation is a common feature of cancers [144]. Preclinical studies of Nrp1 including antibody blockades and genetic knockout models have been shown to reduce tumor growth and progression in prostate, lung, and skin cancers [140].

Properdistatin, a new peptide generated from the plasma protein properdin, inhibited angiogenesis in A-07 human melanoma xenografts, according to Wu et al. [22].

Liu and Miao also demonstrated that the CycMSH peptide linked with Tc-99 m can target metastatic melanoma, implying that CycMSH might be used to identify metastatic melanoma [145]. The peptides produced from the melanocortin 1 receptor could induce cytotoxic T-lymphocyte (CTL) responses to destroy melanoma cells, according to Gonzalez et al. [146]. Peptides for the treatment of kidney cancer have also attracted a lot of attention.

Melanocortin receptors (MC1R-MC5R) are members of the GPCR family. Normal human skin cells, such as keratinocytes and melanocytes, express MC1R, which is primarily involved in skin pigmentation [147].

In melanomas and the majority of human metastatic melanoma-derived tumor cells, MC1R has the highest expression. Alpha-melanocyte-stimulating hormone (MSH) is a 13-AA (1SYSMEHFRWGKPV13) peptide with high affinity for MC1R [148].

## Peptide-based strategies for targeted lung cancer

Worldwide, lung cancer is one of the leading causes of death due to cancer [149, 150]. Histopathologically, lung cancer can be categorized as non-small-cell lung carcinoma (NSCLC; 84%) and small-cell lung carcinoma (SCLC; 15%), with the latter being further subdivided into squamous cell carcinoma (SCC), large cell carcinoma (LCC), and adenocarcinoma [151–154].

Presently, the drugs available for treatment, including crizotinib or erlotinib, are tyrosine kinase inhibitors (TKIs) for cancer treatment (primarily lung adenocarcinoma), exhibiting EGFR mutations or EML4-ALK fusions. However, there are potential inhibitors of SCC that target aberrations in cells; for example, mutated SOX2, amplified FGFR, and mutated DDR2 are currently being investigated in clinical trials [155]. Unfortunately, acquired resistance usually develops within 9–12 months of treatment with these TKIs [156, 157]. To date, no tailored targeting agent has been developed for LCC owing to its lack of consistent genetic alterations. Pemetrexed, a cytotoxic agent approved for treating non-SCC, is the current first-line treatment for LCC [158]. There effective targeted therapies for SCLC other than cisplatin and etoposide. Generally, SCLC and NSCLC have low 5-year survival rates, 7% and 21%, respectively [154], and there are no druggable or effective cell-surface markers for LCC or SCLC. In recent years, for the development of diagnostics and targeted drug delivery systems (TDDS) for both SCLC and NSCLC, researchers have attempted to develop broad-spectrum lung cancer-targeting peptides by biopanning LCC, an undifferentiated cell type. Chi et al. [154] suggested that targeting peptides that bind to undifferentiated LCC cells are also capable of binding to other lung cancer subtypes. Despite the fact that peptide ligands are relatively flexible and have a lower binding affinity to receptors compared with antibodies, the use of peptides in theranostic nanomedicine offers advantages because they are smaller, have reduced immunogenicity, have high multivalency, penetrate deep into tumors, are simpler to synthesize, and are cost-effective in production.

## Peptide-based strategies for targeted gastric cancer

Despite the decreased prevalence and mortality rates of gastric cancer globally in recent years, incidence rates of gastric cancer remain high, notably in Eastern Europe, Eastern Asia, and South America. It has also been demonstrated that the average age at which gastric cancer develops is gradually getting younger [159, 160]. As a conventional treatment for advanced, recurrent, or unresectable gastric cancer, chemotherapy, including cisplatin, docetaxel, 5-fluorouracil, and S-1, has a poor clinical prognosis [161]. Since Provenge (sipuleucel-T) was approved by the FDA as the first cancer vaccine for prostate cancer, peptide vaccine therapy has been widely used for other cancers, including gastric, colorectal, and pancreatic [162]. In recent years, peptide-based vaccines have been tested in patients with gastric cancer, including vascular endothelial growth factor receptor 2- (VEGFR2-) 169, VEGFR1-1084, and lymphocyte antigen 6 complex locus K (LY6K-177) epitope peptides [163, 164]. Tumor angiogenesis can be inhibited with a peptide vaccine targeting VEGFR1. Combinational therapy of a VEGFR1 and VEGFR2 peptide-based vaccine with S-1 plus cisplatin provided higher clinical efficacy, and no severe adverse effects were reported in patients with advanced gastric cancer. As a result of this combinational therapy, the median overall survival time was 14.2 months and the median progression-free survival time was 9.6 months. In comparison with the S-1 plus cisplatin treatment, the median overall survival time was 13 months and the median progression-free survival time was 6 months [163].

## Peptide-based strategies for targeted genitourinary cancers

In recent years, there have been many advances in treatment options for genitourinary malignancies. Among the malignancies found in the genitourinary (GU) system, renal cell carcinoma (RCC) and urothelial carcinoma (UC) benefit most from immune-checkpoint inhibitors (ICIs) alone or in combination with other ICIs or tyrosine kinase inhibitors (TKIs). Nevertheless, an immunologically “cold” and immune-suppressive TME associated with prostate cancer (PCa) has limited the effectiveness of ICIs [165].

In recent years, peptide vaccination has been examined for various cancer subtypes. A novel vaccine strategy, personalized peptide vaccination (PPV) includes administering specially designed HLA-matched peptides to individuals on the basis of their pre-vaccine immunity [166]. Although PPV was considered an attractive strategy for PCa, and even after successful phase II trials, the phase III trial of 310 patients with mCRPC receiving docetaxel found no survival advantage over PBO (HR 1.04; 95%, CI 0.80–1.37, *p* = 0.77) [167–170]. However, even when combined with chemotherapy, personalized vaccination did not result in significant survival differences, as demonstrated in the treatment of CRPC with personalized autologous dendritic-cell-based cancer vaccine (DCvac) plus docetaxel [171]. Ongoing phase III trials are comparing docetaxel + DCvac with docetaxel + PBO for the first-line treatment of mCRPC (NCT02111577). In sum, it seems that more investigations are required to design effective PPVs for genitourinary cancers (Table 1).

**Table1:** Anticancer peptide function in four different cancers: breast, glioma, colorectal, and melanoma

Cancer	Target, group	Peptide name	Function	Refs.
Breast cancer	Tumor suppressor	INK4 family (p16INK4a p15INK4b p18INK4c p19INK4d)	Using the proteins Cdk4 or Cdk6 to inhibit the action of cyclin D and block the progression of the cell cycle	[172]
		CIP/KIP family (p21cip1/waf1 p27kip1 p57 kip2)	CYCLIN–CDK complexes indirectly repress transcription	[173]
		p16 (p16^INK4a^)	Slowing down the progression of the cell cycle by inactivating Cdk4/6	[174]
		p21 (CIP1/WAF1)	Cell cycle arrest is caused by blocking the activity of cyclin–CDK2, cyclin–CDK1, and cyclin–CDK4/6 complexes Competing for PCNA binding with DNA polymerase-δ, hence directly inhibiting DNA synthesis	[175]
		p44	Activating p53 by phosphorylating serine 15, resulting in activating p21 protein, and thus inhibiting cell cycle progression	[176]
		BRCA1	Involved in repairing damaged DNA	[177]
	Cell death provoker	KCCYSL	Imaging the HER2-positive tumors with k_d_ of 295 nM	[178]
		BP*-*FFVLK-YCDGFYACYMDV	Once bound to the cell surface HER2, it transforms into nanofibrillar (NF) structures	[179]
		Bcl-2 family (Bax, Bak, and Bok)	Binding with interactions regulating mitochondrial outer membrane permeabilization, thereby releasing intermembrane space proteins	[180]
		CT20	Promoting mitochondrial aggregation and cytoskeletal disruption	[181]
		RRM-MV	Decreasing BCL-XL and d MCL-1, increasing Bax	[182]
		VDAC1	Interacting with anti-apoptotic proteins	[183]
	Cytolytic agents	RGD	Acting as an inhibitor of integrin–ligand interactions Promoting cell adherence to the matrix, preventing cell apoptosis, and accelerating new tissue regeneration	[184]
		Pleurocidin family (NRC-03 and NRC-07)	Using membranolytic mechanisms to kill breast cancer cells	[185]
		L-K6	Interacting with MCF-7 cells, owing to negatively charged phosphatidylserine thereof	[186]
		p28 peptide	Acting as a cancer-specific antiproliferative agent	[187]
		SKACP003	Inducing dose-dependent cytotoxicity in all cell lines via hydrogenic interaction of the peptide with β-catenin	[179]
	Peptide vaccine	E75	Eliciting a robust anti-HER2 immune response	[188]
Glioma	Cell-penetrating peptides	PepFect14 Transportan10 PepFect3 PepFect28 SynB3 Stearyl-SynB3	Delivery of splice-correcting oligonucleotides (SCOs) to the HeLa pLuc705 cell line Delivering a large protein such as an antibody, oligonucleotide, or colloidal gold as cargo	[189] [190] [191]
	MDGI/FABP3 targeting peptide	CooP (CGLSGLGVA)	Recognizing tumor vessels and invading tumor satellites by interacting with MDGI/H-FABP/FABP3 in brain tumor tissue	[192]
	Transferrin receptor	T7 (HAIYPRH)	Targeting transferrin receptors (TfRs) in a tumor-targeting nanodrug delivery system to enter cells easily without damaging the cells with transferrin (Tf)	[193]
		T12 (THRPPMWSPVWP)	Binding to a different site on TfRs compared with transferrin	[194]
	LRP targeting peptides	LRP-1	As a cellular penetration facilitator, uPA inhibits the activity of extracellular proteases that activate uPA	[195]
		LRP-2	Mediating endocytosis of ligands leading to degradation in lysosomes or transcytosis	[196]
	Interleukin-4 receptor	CRKRLDRNC	IL-4Ra-binding	[197]
Colorectal cancer		PepT 1 (SLC15A1)	Facilitating the cellular translocation of dipeptides and tripeptides and the absorption of numerous peptidomimetic drugs	[198]
		HNP1–3	Mediating lysis of tumors in a concentration-dependent manner Inducing the chemokine interleukin-8 (IL-8) and modulating cytokine expression, thereby modulating immune response and inflammation	[199]
		TCP-1	Delivering fluorescein and anticancer medicines into CRC tissue	[200]
		EphA2-derived peptide	EphA2-specific CTL	[201]
		ABT-737	Inhibition of anti-apoptotic Bcl-2 family	[201]
		Multipeptide cocktail: epitomes of HER2, MVF, GMP, and *n*-MDP	Inhibition of EGF-2	[201]
		Endoglin	Inhibition of angiogenesis	[201]
		CEA691	Induction of tumor-specific CTLs	[201]
		CEA526–533, NP52–59	Activation of tumor-specific CTLs	[201]
Melanoma		M2pep	Binding preferentially to murine M2 macrophages and M2-like TAMs	[202]
		LyP-1	Creating reagents that can specifically destroy tumor lymphatics could be one way to develop the treatment	[203]
		CycMSH	Providing a large surface area for interacting with the target	[204]
		RGD-SSL-Dox	Facilitating the DOX uptake into melanoma cells by integrin-mediated endocytosis	(205)

## Conclusion

Peptide therapy has a number of benefits and drawbacks. Several advantages of peptides, including in vivo stability, proper diffusion into tumor tissue, and low production costs, make them preferable relative to antibiotics, radiotherapy, and chemotherapy in cancer treatment. Three main sources of peptide, that is, natural or bioactive peptides, engineered peptides, and chemical-library-derived peptides, can be applied in diagnosis, prognosis, and prediction of several types of tumor, including breast, glioma, colon, and melanoma.

In this regard, peptide-based strategies for cancer therapy can be designed for a specific target. The peptides can be designed for transcription factors, specific receptors, and tumor suppressor proteins to disrupt the regulation of timing/rate of gene expression and cell cycle progression.

Moreover, some of the designed therapeutic peptides promote cancerous cell apoptosis via inhibiting and activating the anti-apoptotic and pro-apoptotic proteins, respectively. In this line, to directly recognize cancer cells and achieve higher cell penetration, therapeutic peptides can be attached to cytolytic peptides with cationic and amphiphilic properties. Some usage-limiting properties of the designed peptides, including low selectivity and tumor cell uptake, poor tumor permeability and passing through the BBB in glioma cancer, and low plasma stability, can be improved by several techniques, such as intracellular delivery via the intracellular pathway, ligand-mediated tumor targeting, and nanoparticle-based drug delivery systems.

Cancer vaccines based on peptides present tumor antigen epitopes to T cells in a novel and versatile way to induce cell-mediated immunity against cancer. An immune response and, ultimately, T-cell-mediated killing of cancer cells are triggered when the tumor antigens are displayed on the surfaces of cancer cells.

To enhance the immunogenicity of peptide-based cancer vaccines, conjugates and polymers can be used to target peptides to specific immune cells or to include stimulation molecules.

Peptide vaccines have been studied in combination with chemotherapy agents and drugs not intended to treat cancer, with promising results. Another approach involves combining peptide vaccines with highly customizable vaccine carriers, including adjuvants, targeting motifs, and CD4^+^ and CD8^+^ epitopes for complete vaccines. Overall, peptide-based cancer vaccines fail to reach high efficacy levels when used alone, but they show promise when used in combination with other treatments.

Ideally, peptide-based cancer vaccines should be used in combination therapy as a viable treatment option. The development of personalized peptide vaccines is becoming increasingly important as vaccines become custom-tailored to individual patients. Currently, designing and producing personalized peptide vaccines is a time-consuming and costly process, but with new sequencing technologies, bioinformatics, and techniques for predicting T-cell epitopes, this may become a highly valuable tool in the future. The purpose of this study was to illustrate how challenging it is to design a peptide-based cancer vaccine and to balance delivery method, half-life, epitope selection, and immunogenicity to produce a successful vaccine strategy. Despite the fact that the majority of the studies cited in this review were preclinical or at the early stages of clinical studies, there are many ongoing studies on peptide-based cancer vaccines. The ultimate goal of future studies should be to combine CD4^+^ and CD8^+^ responses in vivo to activate DCs and T cells for a long period of time. To be considered successful, no antibodies will need to be customized, multifaceted, or targeted to an individual’s neoantigen repertoire, while also overcoming or reducing the immunosuppressive burden of the tumor microenvironment.

## Data Availability

The datasets used and/or analyzed during the current study are available from the corresponding author on reasonable request.

## Abbreviations

AA: Amino acids
CPPs: Cell-penetrating peptides
MDP: Mycobacteria-derived muramyl dipeptide
DCIS: Ductal carcinoma in situ
IDC: Invasive ductal carcinoma
IBC: Inflammatory breast cancer
TFs: Transcription factors
bHLH: Basic helix–loop–helix
HER2: Human epidermal growth factor receptor 2
BIRD-2: Bcl-2 IP3 receptor disrupter-2
DDS: Drug delivery system
APCs: Antigen-presenting cells
BBB: Blood–brain barrier
CMCSN: Carboxymethyl chitosan nanogel
CRC: Colorectal cancer
cAMP: Cyclic adenosine monophosphate
CREB: CAMP-responsive element-binding protein
Nrp1: Neuropilin-1
CTL: Cytotoxic T-lymphocyte
